# Evaluation of the input site and characteristics of the antegrade fast pathway based on three-dimensional bi-atrial stimulus-ventricle mapping

**DOI:** 10.1007/s10840-021-01026-7

**Published:** 2021-07-07

**Authors:** Kazuhisa Matsumoto, Takeshi Tobiume, Tomomi Matsuura, Takayuki Ise, Kenya Kusunose, Koji Yamaguchi, Shusuke Yagi, Daijyu Fukuda, Tetsuzo Wakatsuki, Hirotsugu Yamada, Takeshi Soeki, Masataka Sata

**Affiliations:** Department of Cardiology, Tokushima University Hospital, 3-18-15 Kuramoto-cho, Tokushima City, Tokushima 770-8501 Japan

**Keywords:** Antegrade fast pathway, St-V map, St-H map, Left atrial input, Right atrial input

## Abstract

**Purpose:**

Previous studies examined the right atrial (RA) input site of the antegrade fast pathway (AFp) (AFpI). However, the left atrial (LA) input to the atrioventricular (AV) node has not been extensively evaluated. In this study, we created three-dimensional (3-D) bi-atrial stimulus-ventricle (St-V) maps and analyzed the input site and characteristics of the AFp in both the RA and LA.

**Methods:**

Forty-four patients diagnosed with atrial fibrillation or WPW syndrome were included in this study. Three-dimensional bi-atrial St-V mapping was performed using an electroanatomical mapping system. Sites exhibiting the minimal St-V interval (MinSt-V) were defined as AFpIs and were classified into seven segments, four in the RA (F, S, M, and I) and three in the LA (M1, M2, and M3). By combining the MinSt-V in the RA and LA, the AFpIs were classified into three types: RA, LA, and bi-atrial (BA) types. The clinical and electrophysiological characteristics were compared.

**Results:**

AFpIs were most frequently observed at site S in the RA (34%) and M2 in the LA (50%), and the BA type was the most common (57%). AFpIs in the LA were recognized in 75% of the patients. There were no clinical or electrophysiological indicators for predicting AFpI sites.

**Conclusions:**

Three-dimensional bi-atrial St-V maps could classify AFpIs in both the RA and LA. AFpIs in the LA were frequently recognized. There were no significant clinical or electrophysiological indicators for predicting AFpI sites, and 3-D bi-atrial St-V mapping was the only method to reveal the precise AFp input site**.**

## Introduction

The exit site of the retrograde fast pathway (RFp) from the atrioventricular node (AVN) can be determined by mapping the earliest activation site in the atrium during constant pacing from the ventricle. On the other hand, the input site of the antegrade fast pathway (AFp) to the AVN has been very difficult to determine and only stimulus-His (St-H) mapping can identify the input site of the AFp to the AVN (AFpI) [1–5]. The use of a three-dimensional (3-D) mapping system makes identification of the exact location of the AFpI easier to determine [4, 5]. Previous studies, however, have examined the AFpI only in the right atrium (RA) (AFpI[RA]). The left atrial (LA) input to the AVN has not been extensively evaluated and only Gonzalez et al. have suggested that an AFpI exists in the LA (AFpI[LA]) with high probability in 95% of patients [6]. In this study, we created 3-D bi-atrial stimulus-ventricle (St-V) maps, which were equivalent to St-H maps, and analyzed the input site and characteristics of the AFp in both the RA and LA.

## Methods

### Study population

From June 1, 2019, to August 31, 2020, forty-four consecutive patients (31 men and 13 women, 64 ± 14 years, range 25 to 80 years) with atrial fibrillation (AF) (18 paroxysmal and 16 persistent) or Wolff-Parkinson-White (WPW) syndrome with a left free wall accessory pathway who underwent catheter ablation were included in this study. Patients with first degree atrioventricular block and left bundle branch block were excluded because an AFp might not exist in the former and because transient AV block during mapping might occur in the latter. The institutional review board of Tokushima University approved the study protocol. Written informed consent was obtained from all patients.

### Electrophysiological study

Patients with atrial fibrillation were studied under deep sedation with a continuous infusion of propofol. They were on a ventilator and underwent continuous monitoring of the blood pressure, oxygen saturation, and bispectral index. Patients with WPW syndrome were studied under local anesthesia using xylocaine. Venous access was established percutaneously from the right jugular vein and right femoral vein to introduce electrode catheters into the RA, right ventricle (RV), coronary sinus (CS), and LA. A His bundle electrogram recording catheter (His) was used in the case of WPW syndrome, but not in the case of AF. All patients required a transseptal left heart catheterization for a pulmonary vein isolation or accessory pathway ablation. To record the electrocardiogram from the RA and CS, a 6-Fr catheter with 20 electrodes (BeeAT; Japan Lifeline, Tokyo) was inserted via the right jugular vein into the CS. A 5-Fr catheter with 5 electrodes (Arma, Century Medical Inc., Tokyo, Japan) was positioned in the RV apex (RVA). The CARTO®3 System (Biosense Webster, Irvine, California) was used for 3-D mapping in all patients. The bipolar electrocardiograms were filtered at 30–400 Hz for the electrophysiological analysis (CardioLab, GE Healthcare Japan, Tokyo).

### Identifying the input site of the antegrade fast pathway using 3-D bi-atrial St-V maps

**Fig. 1:**
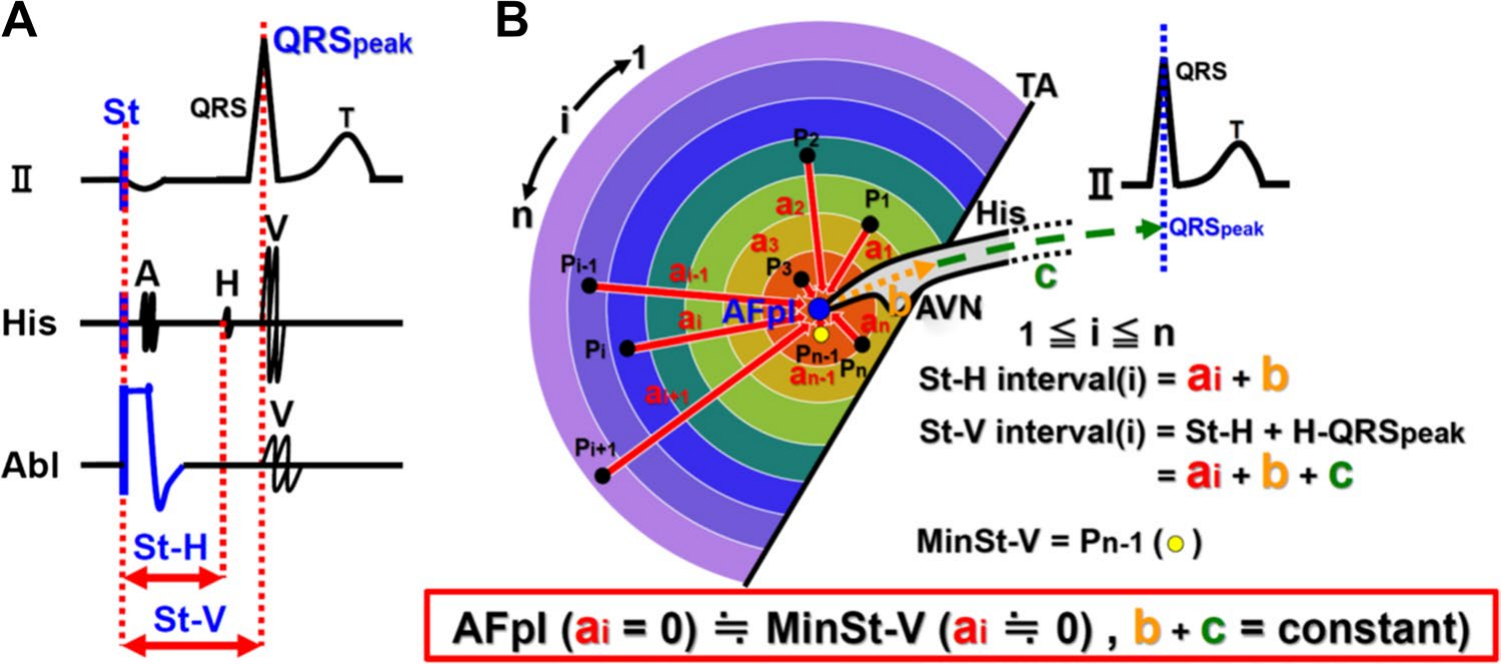
Schematic presentation of the method of St-V mapping (intracardiac electrogram (A) and electroanatomical map (B)). (**A**) a: During constant pacing from Abl catheter, St-V interval, which means the conduction time between the stimulation artifact and the peak of QRS wave in lead II, was measured at each pacing site. Pacing output was set at 3–4 V/1 ms (**B**) St-V interval of the *i*th pacing site was shown as “ai + b + c.” At MinSt-V, ai was approximately zero and MinSt-V was almost identical with AFpI, where ai was equal to zero. His, His bundle catheter; Abl, ablation catheter; St, stimulus; A, atrial electrogram; H, His bundle electrogram; V, ventricular electrogram; St-H, stimulus-His; St-V, stimulus-ventricle; AVN, atrioventricular node; AFp, antegrade fast pathway; AFpI, input site of AFp to AVN; Pi, *i*th pacing site; Pn, *n*th pacing site; St-H interval(i), St-H interval of the *i*th pacing site; St-V interval(i), St-V interval of the *i*th pacing site; MinSt-V, the pacing site showing the minimal St-V interval; a, conduction time between the pacing site and AFpI; b, conduction time between AFpI and His bundle electrogram; c, conduction time between His bundle electrogram and the peak of QRS wave in lead II

During the waiting period after the ablation, 3-D bi-atrial St-V maps for identifying the AFpI were created in all patients during constant atrial pacing at 100 ppm (600 ms) from a 7-Fr irrigation catheter (Thermocool SmartTouch SF, Biosense Webster). The pacing output was set at 3–4 V/1 ms in order to avoid capturing the AVN, His bundle, and ventricles. As shown in Fig. 1, constant pacing was performed at one pacing site (Pi) and, after reaching a steady state, the interval between the stimulation artifact and peak of the QRS wave in lead II (St-V interval) was measured. Data regarding both the location of the pacing site (Pi) and St-V interval at the Pi were stored on the 3-D map. Then, the pacing catheter was moved to the next site (Pi + 1). The AFpI was defined as the site exhibiting the minimal St-V interval (MinSt-V). The AFpI[RA] and AFpI[LA] were defined as the MinSt-V in the RA (MinSt-V[RA]) and LA (MinSt-V[LA]), respectively. The sites where the His bundle electrocardiogram was recorded were also stored on the 3-D map during sinus rhythm to identify the apex of the triangle of Koch (TOK) without the electrocardiographic information, and the A-H interval during sinus rhythm was measured at those sites. The anatomical information related to the roof and floor of the CS ostium, tricuspid annulus, and mitral annulus were also stored on the 3-D map.

### Classification of the input site of the antegrade fast pathway

**Fig. 2:**
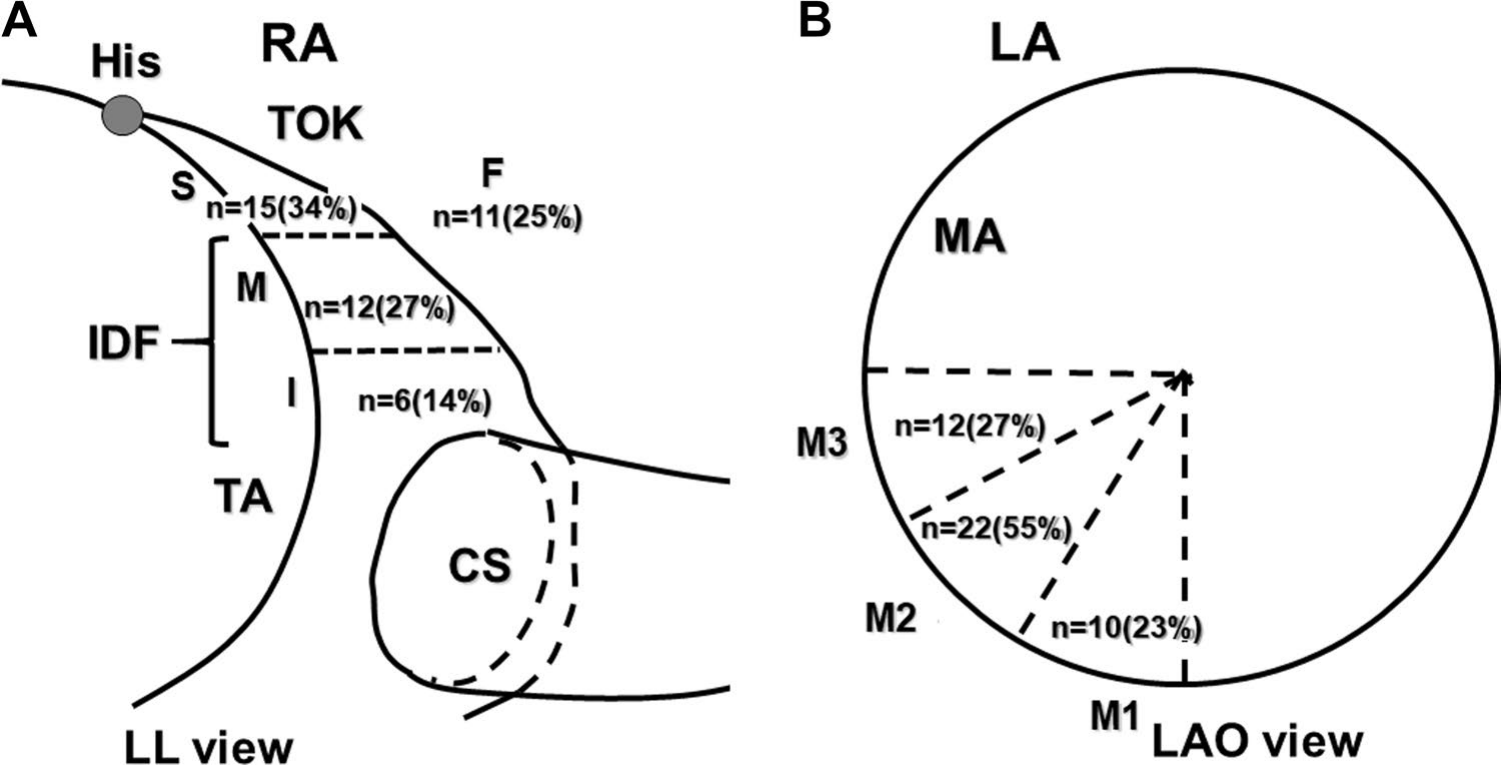
Schematic presentation of the triangle of Koch (TOK) of the right atrium (RA) in the left lateral (LL) view (A) and the mitral annulus (MA) of left atrium (LA) in the left anterior oblique view (B). **A**: TOK was divided into 3 parts (superior third part, middle third part, and inferior third part were defined as site S, site M, and site I, respectively), and a part posterior to the TOK was defined as site F. **B**: MA was divided into 3 parts. The direction of 6–7, 7–8, and 8–9 o’clock of MA were defined as site M1, site M2, and site M3, respectively. His, His bundle electrogram recording site; TOK, triangle of Koch; IDF, inferiorly dislocated AFp

As shown in Fig. 2, for the classification of the AFpI, the atrial septum was subdivided into seven segments, four on the RA aspect and three on the LA side. In the RA, the TOK was classified into three equidistant parts: superior, middle, and inferior thirds defined as sites S, M, and I, respectively. Also, the part posterior to the TOK was defined as site F. If the AFpI was located at site M or I, the AFpI was defined as an inferiorly dislocated AFp (IDF). In the LA, the AFpI was classified into three segments along the mitral annulus (MA): the 6–7 o’clock, 7–8 o’clock, and 8–9 o’clock directions along the mitral annulus were labeled as sites M1, M2, and M3, respectively.

### Classification of the type of input site of the antegrade fast pathway by combining the right and left atria

**Fig. 3:**
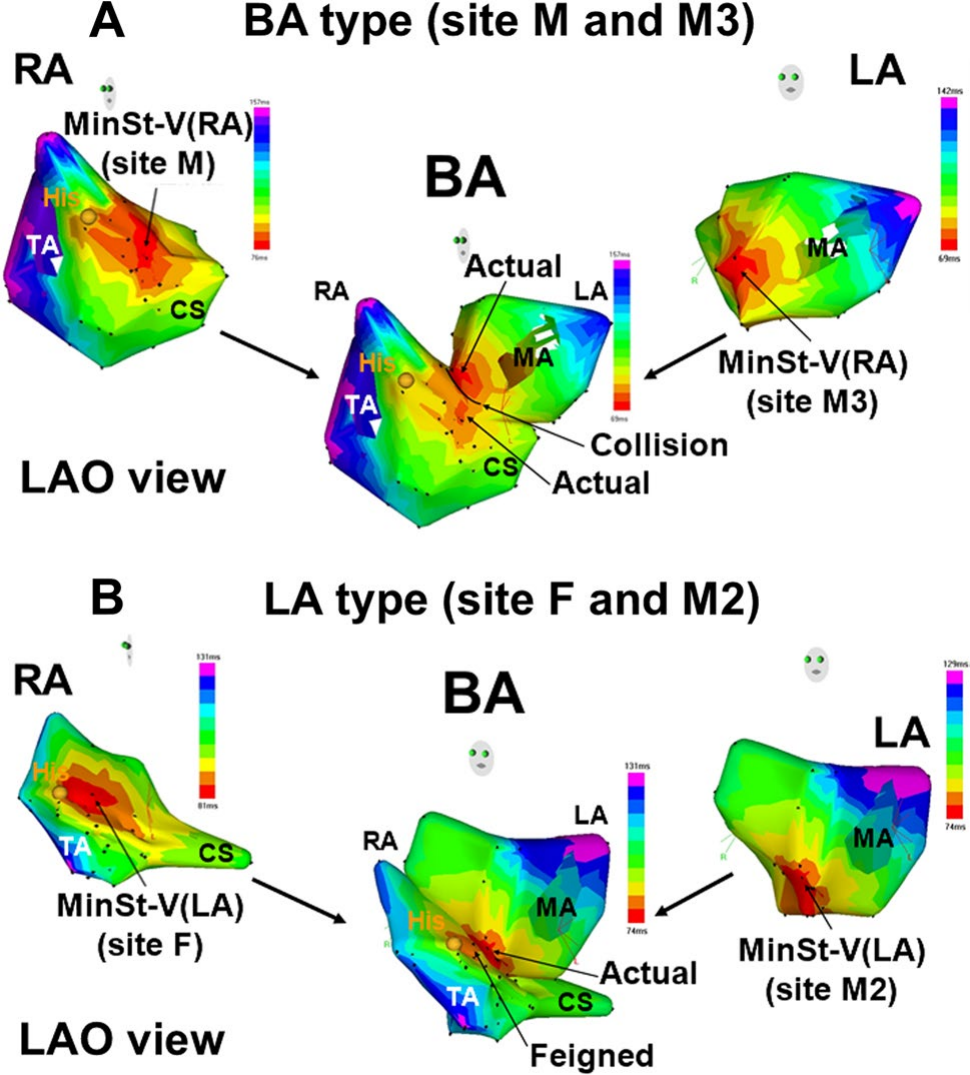
Examples of AFpI of BA type (A) and LA type (B) using 3-D bi-atrial St-V map. **A**: In BA type of AFp, minimal St-V interval in RA and LA showed almost the same value. MinSt-V(RA) and MinSt-V(LA) were clearly separated with some sites on the shortest distance between MinSt-V(RA) and MinSt-V(LA) along the atrial septum where St-V interval was longer than both minimal St-V intervals in RA and LA. In BA type, both MinSt-V(RA) and MinSt-V(LA) represent two different AFpI (actual). **B**: In LA type of AFp, minimal St-V interval in LA showed smaller value than that in RA and there was no site on the shortest distance between MinSt-V(RA) and MinSt-V(LA) along the atrial septum where the St-V interval was longer than minimal St-V interval in RA. In LA type, MinSt-V(LA) represents the only AFpI (actual) while MinSt-V(RA) does not represent an AFpI (feigned)

As shown in Fig. 3, by combining the AFpI[RA] (MinSt-V[RA]) and AFpI[LA] (MinSt-V[LA]) for the classification of the type of AFpI, the AFpI was classified into three types: RA type, LA type, and bi-atrial (BA) type. The RA type was defined when the following two conditions were met: (1) the MinSt-V[RA] was shorter than that in the LA, and (2) there was no site on the shortest distance between the MinSt-V[RA] and MinSt-V[LA] along the atrial septum where the St-V interval was longer than the MinSt-V[LA]. In the RA type, the MinSt-V[RA] was the only AFpI (actual) and the MinSt-V[LA] was not the AFpI (feigned). An LA type was defined when the following two conditions were met: (1) the MinSt-V[LA] was shorter than that in the RA, and (2) there were no sites on the shortest distance between the MinSt-V[RA] and MinSt-V[LA] along the atrial septum where the St-V interval was longer than the MinSt-V[RA]. In the LA type, the MinSt-V[LA] was the only AFpI (actual) and the MinSt-V[RA] was not the AFpI (feigned). A BA type was defined when the following two conditions were met: (1) the MinSt-V[RA] and MinSt-V[LA] exhibited almost the same value, and (2) there were some sites on the shortest distance between the MinSt-V[RA] and MinSt-V[LA] along the atrial septum where the St-V interval was longer than both the MinSt-V[RA] and MinSt-V[LA]. In the BA type, the MinSt-V[RA] and MinSt-V[LA] were two separate AFpIs (actual).

### Statistical analysis

Data are expressed as the mean ± SD. Differences between the means were compared using a one-way analysis of variance or the Kruskal–Wallis test. A Levene’s test was used to check for the equality of variance. The differences in the proportions were compared using the chi-square test. A *P* < 0.05 was considered statistically significant. All statistical analyses were performed with the Statistical Package for the Social Sciences for Windows software (SPSS, version 27, Chicago, IL, USA).

## Results

### Clinical and electrophysiological characteristics of the study population

As shown in Table 1, 66% (29/44) of the patients in this study population continued antiarrhythmic drugs during the procedure. In patients with WPW syndrome, the antiarrhythmic drugs were discontinued preoperatively. Twenty-nine of 34 patients with AF continued on antiarrhythmic drugs. Class I drugs were used in 13 patients (cibenzoline 7, pilsicainide 5, and propafenone 1), class II (bisoprolol) in 7 patients, and class IV in 9 patients (verapamil 4 and bepridil 5). No class III drugs were used. The mean left atrial volume index (LAVI) was 40 ± 14 mL/m^2^. The A-H interval during sinus rhythm was 94 ± 16 ms. The number of mapping points was 29 ± 9 points in the RA and 25 ± 10 points in the LA. The minimal St-V interval was 162 ± 45 ms in the RA and 161 ± 42 ms in the LA.

**Table 1:** Clinical and electrophysiological characteristics of the study population

	*n* = 44
Age (years) (mean ± SD)	64 ± 14
Male/female ratio	31/13
Type of arrhythmia	
Paroxymal AF/persistent AF/WPW syndrome	18/16/10
Continuation of antiarrhythmic drugs (*n* (%))	29 (66)
Vaughan Williams classification (I/II/III/IV)	13/7/0/9
LAVI (ml/m^2^) (mean ± SD)	40 ± 14
A-H interval during sinus rhythm (ms) (mean ± SD)	94 ± 16
Number of mapping points (mean ± SD)	
RA/LA	29 ± 9/25 ± 10
Minimal St-V interval (mean ± SD)	
RA/LA	162 ± 45/161 ± 42
Classification of the AFp (RA type/LA type/BA type)	11/8/25
Site of the minimal St-V interval in the RA (S/M/I/F)	15/12/6/11
Site of the minimal St-V interval in the LA (M1/M2/M3)	10/22/12

*SD*, standard deviation

### Location of the input site of the antegrade fast pathway in the RA

**Fig. 4:**
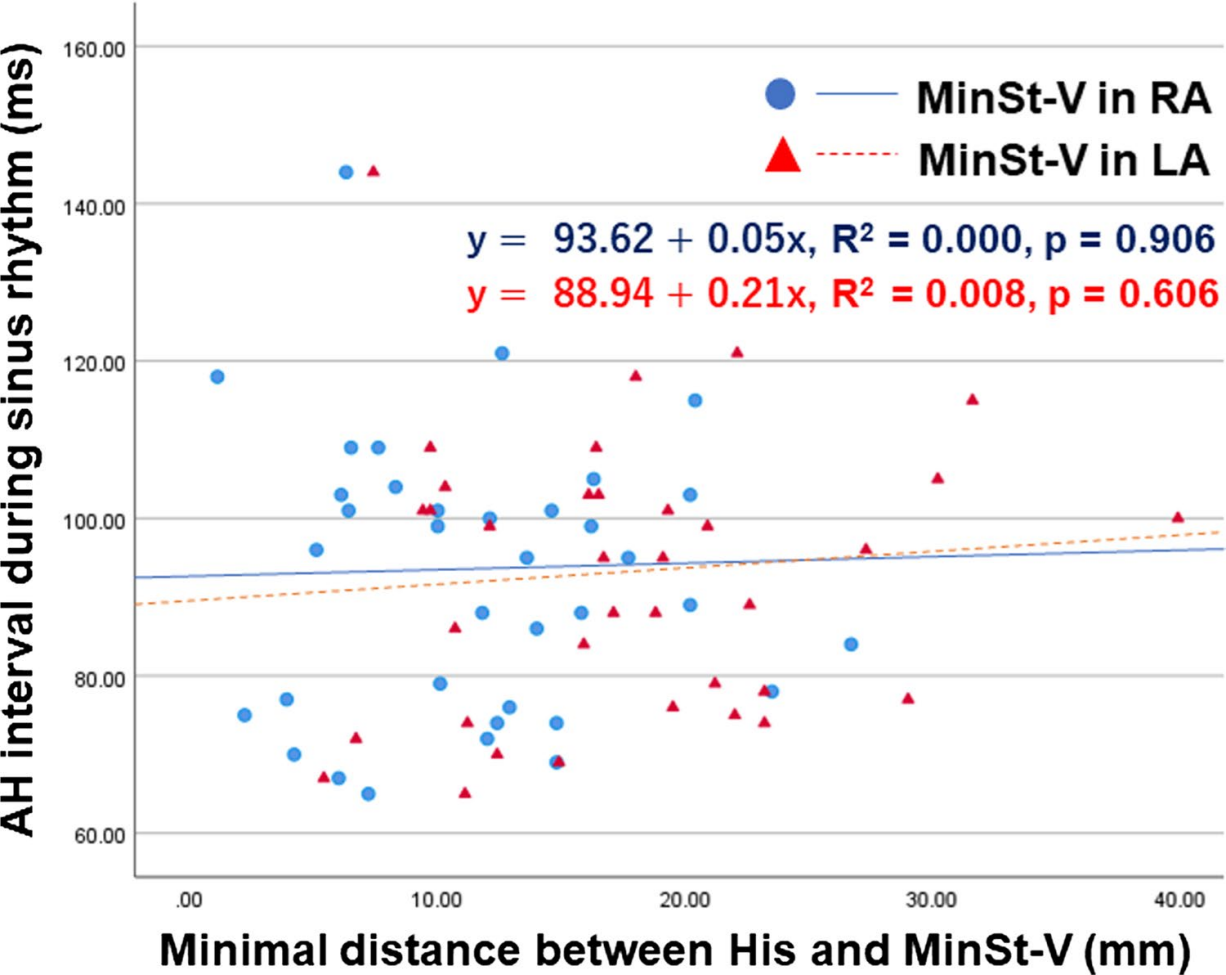
Relationship between A-H interval during sinus rhythm and the minimal distance between His and AFpI(MinSt-V) in RA. There was no relationship between A-H interval during sinus rhythm and the minimal distance between His and AFpI (MinSt-V) in RA and LA. *p* = 0.906 (RA), *p* = 0.606 (LA)

As shown in Fig. 2 and Table 2, the MinSt-V[RA] was observed at site F in 11 patients (25%), site S in 15 (34%), site M in 12 (27%), and site I in 6 (14%). An IDF was observed in 41% of the patients. There were no significant differences among these four groups with regard to the age, gender, type of arrhythmia, LAVI, A-H interval during sinus rhythm, minimal St-V interval, and site of the MinSt-V[LA]. The minimal distance between the His bundle electrogram recording site and MinSt-V[RA] did not correlate with the A-H interval during sinus rhythm (Fig. 4).

**Table 2:** Clinical and electrophysiological characteristics of the sites showing the minimal St-V interval in the RA

		Site F (*n*=11)	Site S (*n*=15)	Site M (*n*=12)	Site I (*n*=6)	*p* value
Age (years) (mean ± SD)		69±7	67±9	64±14	49±28	0.112
Male/female ratio		8/3	9/6	9/3	5/1	0.703
Type of arrhythmia						
Paroxysmal AF/persistent AF/WPW syndrome		3/6/2	7/6/2	6/4/2	2/0/4	0.117
LAVI (ml/m^2^) (mean ± SD)		48±17	39±11	37±15	28±6	0.155
A-H interval during sinus rhythm (ms) (mean ± SD)		89±14	96±22	95±14	97±13	0.651
Minimal St-V interval (ms) (mean ± SD)	RA	161±50	174±30	162±46	149±65	0.387
	LA	155±48	170±27	170±42	145±60	0.313
Site of the minimal St-V interval in the LA (M1/M2/M3)		1/7/3	4/7/4	5/3/4	0/5/1	0.242

### Location of the input site of the antegrade fast pathway in the LA

As shown in Fig. 2 and Table 3, the MinSt-V[LA] was observed at site M1 in 10 (23%), site M2 in 22 (50%), and site M3 in 12 (27%). There were no significant differences among these three groups with regard to the age, gender, type of arrhythmia, LAVI, A-H interval during sinus rhythm, minimal St-V interval, and site of the MinSt-V[RA]. Interestingly, only sites F and S were recognized in the LA type. The minimal distance between the His bundle electrogram recording sites and MinSt-V(LA) was not correlated with the A-H interval during sinus rhythm (Fig. 4).

**Table 3:** Clinical and electrophysiological characteristics of the sites with the minimal St-V interval in the LA

		Site M1 (*n*=10)	Site M2 (*n*=22)	Site M3 (*n*=12)	*p* value
Age (years) (mean ± SD)		66±10	68±12	56±17	0.06
Male/female ratio		9/1	14/8	8/4	0.3
Type of arrhythmia					
Paroxysmal AF/persistent AF/WPW syndrome		5/3/2	7/9/6	6/4/2	0.812
LAVI (ml/m^2^) (mean ± SD)		31±11	45±16	38±12	0.08
A-H interval during sinus rhythm (ms) (mean ± SD)		95±14	98±17	86±15	0.094
Minimal St-V interval (ms) (mean ± SD)	RA	170±42	161±51	157±40	0.547
	LA	166±40	160±50	161±31	0.784
Site of the minimal St-V interval in the RA (F/S/M/I)		1/4/5/0	7/7/3/5	3/4/4/1	0.242

### Type of the input site of the antegrade fast pathway by combining the right and left atria

As shown in Table 3, the AFpI was classified as an RA type in 11 (25%), LA type in 8 (18%), and BA type in 25 (57%). There were no significant differences among these three groups with regard to the age, gender, type of arrhythmia, LAVI, A-H interval during sinus rhythm, minimal St-V interval, site of the MinSt-V[RA], and site of the minimal St-V interval in the LA. Examples of the AFpI in the BA type and LA type are shown in Fig. 3. Figure 3A shows an example of a BA type, and the MinSt-V[RA] was site M and MinSt-V[LA] site M3. The MinSt-V[RA] and MinSt-V[LA] were 76 ms and 69 ms, respectively. There were some sites on the shortest distance between the MinSt-V[RA] and MinSt-V[LA] along the septal wall where the St-V interval was longer than both the MinSt-V[RA] and MinSt-V[LA], and the 3-D bi-atrial St-V map exhibited two clearly separate AFpIs. Figure 3B shows an example of an LA type, and the MinSt-V[RA] was site F and MinSt-V[LA] site M2. The MinSt-V[RA] and MinSt-V[LA] were 81 ms and 74 ms, respectively. There was no site on the shortest distance between the MinSt-V[RA] and MinSt-V[LA] along the atrial wall where the St-V interval was longer than the MinSt-V[RA], and the 3-D bi-atrial St-V map exhibited only one AFpI.

## Discussion

### Major findings

In the present study, using the 3-D bi-atrial St-V map, it was possible to classify the input sites of the AFp in both the RA and LA. An AFpI[LA] was frequently observed and was recognized in 75% of the patients. The AFpI[LA] and AFpI[RA] were most frequently observed at site M2 (50%) and site S (34%), respectively. The A-H interval during sinus rhythm was not correlated with the minimal distance between the His bundle electrogram recording sites and the MinSt-V[RA] and MinSt-V[LA] (Table 4).

**Table 4:** Clinical and electrophysiological characteristics of the RA, LA, and BA type

		RA type (*n*=11)	LA type (*n*=8)	BA type (*n*=25)	*p* value
Age (years) (mean ± SD)		65±14	70±7	62±15	0.325
Male/female ratio		10/1	4/4	17/8	0.143
Type pf arrhythmia					
Paroxysmal AF/persistent AF/WPW syndrome		4/3/4	4/3/1	10/10/5	0.751
LAVI (ml/m^2^) (mean ± SD)		39±13	37±9	41±16	0.812
A-H interval during sinus rhythm (ms) (mean ± SD)		97±20	104±12	90±15	0.094
Minimal St-V interval (ms) (mean ± SD)	RA	142±48	183±50	165±41	0.148
	LA	157±45	159±46	163±42	0.899
Site of the minimal St-V interval in the RA (F/S/M/I)		1/4/3/0	3/4/0/0	6/7/7/5	0.146
Site of the minimal St-V interval in the LA (M1/M2/M3)		3/5/3	2/6/0	5/11/9	0.366

### St-V map as an alternative to an St-H map

In the previous studies, the St-H map was the standard map used for the determination of the AFpI [1–5]. In this study, an St-V map was used instead of the St-H map because a His catheter was not used in the majority of the patients who had atrial fibrillation (77%) and the St-V map was equivalent to the St-H map. As shown in Fig. 1, the St-V interval (ai + b + c) was equal to the interval that consisted of the St-H interval (ai + b) and interval between the onset of the His bundle electrogram and peak of the QRS wave in lead II (c). Here, the interval between the onset of the His bundle electrogram and peak of the QRS wave in lead II (c) was equivalent to the sum of the H-V interval and ventricular activation time (VAT: the time from the onset of the QRS wave to the peak of the R wave). The H-V interval and VAT are reported to have a very high reproducibility with minimal variability [7–10]. Therefore, the St-V map was considered an alternative to the St-H map.

### Left antegrade fast pathway input to the atrioventricular node

To the best of our knowledge, this study was the first to investigate the detailed input site of the AFp in the LA. The 3-D bi-atrial St-V mapping with the CARTO system in this study revealed that an AFpI[LA] was present in 75% of the patients and was the only AFpI in 18%. In the previous reports, an AFpI[LA] was suggested in cases with a left posteroseptal accessory pathway ablation [11, 12] or left-sided fast pathway ablation for typical atrioventricular nodal reentrant tachycardia (AVNRT) [13]. Moreover, the low success rate (46–69%) of a right-sided fast pathway ablation for AVNRT reported by Mitrani et al. may also suggest the residual presence of an intact AFpI[LA] after an AFpI[RA] ablation [14]. The only report about the probable presence of an AFpI[LA], by Gonzalez et al., suggested that an AFpI[LA] existed with high probability in 95% of the patients [6]. That probability of the presence of an AFpI[LA] was higher than that in this study (75%), which was because atrial extrastimulation to identify the AFpI[LA] was used in their report.

### Right antegrade fast pathway input to the atrioventricular node

The 3-D bi-atrial St-V mapping with the CARTO system in this study revealed the presence of an AFpI[RA] in 82% of the patients and only an AFpI in 25%. An inferiorly dislocated AFp (IDF) was recognized in 41% (18/44) of the patients. The incidence of an IDF in this study was much higher than that reported by Delise et al*.* (10%) [2], but was similar to that reported in the other Japanese studies (33–56%) [3, 4]. These differences might be related to the ethnic differences and therefore, further studies will be needed.

### Clinical and electrophysiological indicators of the input site of the antegrade fast pathway

In this study, the clinical and electrophysiological indicators including the age, gender, type of arrhythmia, LAVI, A-H interval during sinus rhythm, minimal St-V interval, and site of the MinSt-V[RA] had no significant discriminating ability regarding the input sites of the AFp. Therefore, pending further studies, St-V or St-H mapping remains the only method for identifying the precise location of the AFpI.

### Study limitations

1. This study was conducted at a single center and included only a small number of patients.
2. Antiarrhythmic drugs were continued in many patients (66%) because the majority of the patients studied had AF. Therefore, the St-V interval and other electrophysiological parameters could have been affected by those drugs.
3. Two different diseases, AF and WPW, were included in this study, and all AF patients were studied under deep sedation and all WPW patients under local anesthesia. Therefore, the difference in the disease and effect of the sedation could have affected the electrophysiological parameters. Further studies are needed to evaluate the effects of those.
4. In this study, we did not compare the effect of isoprenaline loading. The possibility that the input site of the AFp could change according to the isoprenaline loading remained.
5. We fixed the pacing cycle length at 600 ms in order to evaluate the AFp, to overdrive the sinus rate, and to avoid Wenckebach periodicity. However, keeping a uniform pacing rate might have led us to overlook other concealed AV pathways.
6. In this study, the St-V map was not compared with the St-H map. Although further studies will be needed to confirm this, it is reasonable to assume that the two maps would be equivalent.

## Conclusion

The 3-D bi-atrial St-V map could classify the input site of the AFp in both the RA and LA. An AFpI[LA] was frequently present and was recognized in 75% of the patients. Except for the 3D bi-atrial St-V mapping, there were no significant clinical or electrophysiological indicators that could reveal the precise input site of the AFp.

## Abbreviations

RA: Right atrium/right atrial
LA: Left atrium/left atrial
BA: Bi-atrial
A-H: Atrial-His
H-V: His-ventricle
AFp: Antegrade fast pathway
AFpI: Input site of AFp to the AVN
AFpI(RA): AFpI in the RA
AFpI(LA): AFpI in the LA
IDF: Inferiorly dislocated AFp
Pi: Pacing site
TOK: Triangle of Koch
MA: Mitral annulus
TA: Tricuspid annulus
St-H interval: Interval between the stimulation artifact and His bundle electrogram
St-V interval: Interval between the stimulation artifact and peak of the QRS wave in lead
MinSt-V: The site showing the minimal St-V interval
MinSt-V(RA): MinSt-V in RA
MinSt-V(LA): MinSt-V in LA
VAT: Ventricular activation time (the time from the onset of the QRS wave to the peak of the R wave
